# A Rare Cause of Headache

**DOI:** 10.5811/westjem.2015.10.28635

**Published:** 2016-01-12

**Authors:** Rohat Ak, Fatih Doğanay, Özge E. Onur

**Affiliations:** Fatih Sultan Mehmet Education and Research Hospital, Department of Emergency Medicine, Bostanci/Istanbul, Turkey

## INTRODUCTION

**Figure 1:**
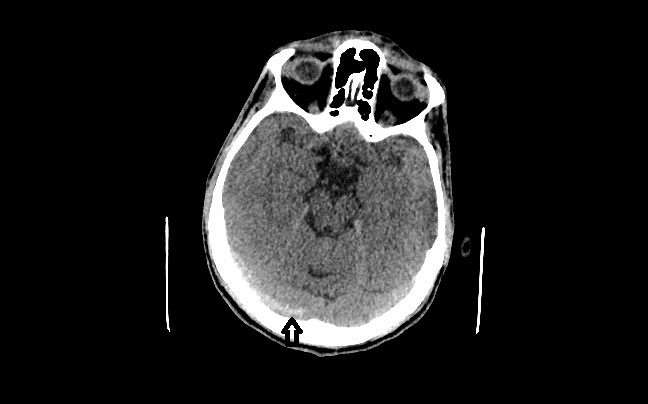
Computed tomography of the brain demonstrating hyperdensity in the region of right transverse sinus (arrow).

**Figure 2:**
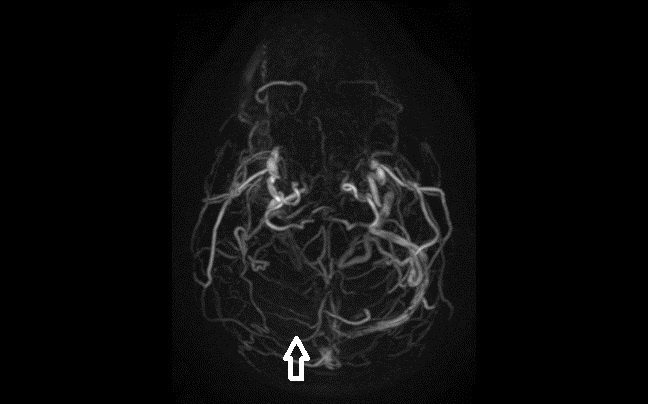
Magnetic resonance venography of the brain demonstrating lack of flow in right transverse sinus (arrow).

A 45-year-old man presented with headache for two days. He described the quality of headache as throbbing, and it was unilateral. There was no history of fever, vomiting, blurred vision, ear discharge or trauma, no relevant past medical or drug history and no family history of note. On examination, he was afebrile with pulse 76/min, regular, blood pressure of 130/80mmHg. His pupils and speech appeared normal. There were no papilledema, sensory deficit, focal neurological deficit or signs of meningeal irritation. Hyperdensity of right transverse sinus (Figure 1) and superior sagittal sinus was identified on unenhanced computed tomography (CT). Magnetic resonance venography (MRV) demonstrated lack of flow in right transverse sinus (Figure 2) and superior sagittal sinus.

## DIAGNOSIS

Cerebral venous sinus thrombosis (CVST) is a rare condition. According to the International Study on Cerebral Vein and Dural Sinus Thrombosis, the most commonly affected site is the transverse sinus, followed by superior sagittal sinus and straight sinus.^1^ Predisposing risk factors may include the following: sinusitis, medications, malignancy, dehydration, prothrombotic conditions, head injury and inflammatory diseases.^2^ Neuroimaging modalities of choice in CVST are CT and magnetic resonance imaging (MRI) with MRV. CT may be normal in 15–30% cases, but MRI with MRV is almost 100% diagnostic.^3^

According to the guidelines of the European Federation of Neurological Societies, the first-line treatment for CVST is antithrombolysis. Our patient was given anticoagulant therapy for six months, after which he had recovered fully.
